# Conceptualizing 20 years of engaged scholarship: A scoping review

**DOI:** 10.1371/journal.pone.0193201

**Published:** 2018-02-28

**Authors:** Marianne Beaulieu, Mylaine Breton, Astrid Brousselle

**Affiliations:** 1 Centre de recherche Charles-Le Moyne—Saguenay–Lac-Saint-Jean sur les innovations en santé, Longueuil, Québec, Canada; 2 Département des sciences de la santé communautaire, Université de Sherbrooke, Longueuil, Québec, Canada; 3 School of Public Administration, University of Victoria, Victoria, British Columbia, Canada; University of Stirling, UNITED KINGDOM

## Abstract

Engaged scholarship, a movement that has been growing steadily since 1995, offers a new way of bridging gaps between the university and civil society. Numerous papers and reports have been published since Boyer’s foundational discourse in 1996. Yet, beyond a growing interest in orienting universities’ missions, we observed a lack a formal definition and conceptualization of this movement. Based on a scoping review of the literature over the past 20 years, the objective of this article is to propose a conceptualization of engaged scholarship. More specifically, we define its values, principles, and processes. We conclude with a discussion of the implications of this new posture for faculty and students, as well as for the university as an institution.

## Introduction

The use and relevance of evidence produced by research are a major concern for researchers and, increasingly, for universities. There are several reasons for this: the impermeable wall between public policies and scientific knowledge [1, 2], the increasingly utilitarian view of the university’s mission [3], and increased scepticism in the public around certain areas of scientific consensus [4], among others.

The researcher’s role has been largely affected by several emerging movements aimed at strengthening the influence of scientific evidence, including knowledge translation [5], participatory research [6], service user involvement [7], and public engagement [8]. At the institutional level, universities are facing questions regarding their mission and their relations with civil society. Ernest Boyer, former president of the Carnegie Foundation, suggested the university should be developing “solutions to the nation's most pressing civic, social, economic, and moral problems”, rather than “being viewed as a place where students get credentialed and faculty get tenured” [9, p.14].

Boyer described the emergence of a “new” stream in the academic world, engaged scholarship (e.g. engaged teaching, engaged research, engaged service), still perceived today as a promising avenue both for increasing the university’s legitimacy and for addressing the knowledge-to-action gap.

“*At one level*, *the scholarship of engagement means connecting the rich resources of the university to our most pressing social*, *civic*, *and ethical problems*.*… Campuses would be viewed by both students and professors not as isolated islands*, *but as staging grounds for action*. *But*, *at a deeper level*, *I have this growing conviction that what’s also needed is not just more programs*, *but a larger purpose*, *a larger sense of mission*, *a larger clarity of direction in the nation's life as we move toward century twenty-one*. *Increasingly*, *I'm convinced that ultimately*, *the scholarship of engagement also means creating a special climate in which the academic and civic cultures communicate more continuously and more creatively with each other*, *… enriching the quality of life for all of us*.” [9, p.19-20].

Boyer’s ideas have quietly permeated the American education sector and, in recent years, have spread into many disciplines and across different countries. This movement has evolved considerably in recent years, but there is, as yet, no exhaustive synthesis of new developments. While academic engagement is not the only movement concerned with democratizing and encouraging the use of scientific evidence, its recent emergence raises the need for in-depth analysis to understand its underlying premises and characteristics, so that it can be defined more clearly. Analysis is needed to delineate its specific attributes and to position it in relation to other movements aimed at strengthening the influence of research-based evidence.

Our objective here is to define and conceptualize engaged scholarship based on a scoping review of the literature. This article will be of interest to professors, researchers, students, and university administrators, not only for the posture we describe, but also for highlighting some promising means of creating constructive channels for exchange with civil society.

## Methods

We conducted a scoping review, which “is a form of knowledge synthesis that addresses an exploratory research question aimed at mapping key concepts, types of evidence, and gaps in research related to a defined area or field by systematically searching, selecting and synthesizing existing knowledge” [10, p.1293-1294]. We followed the five-stage framework proposed by Arksey and O’Malley [11]: 1) identifying the research question; 2) searching for relevant studies; 3) selecting studies; 4) charting the data; and 5) collating, summarizing, and reporting results. To these stages, Arksey and O’Malley [11] add an optional sixth, which involves consulting with stakeholders to inform or validate study findings.

### Identifying the research question

The aim of this scoping review is to define engaged scholarship.

### Searching for relevant studies

The period under review extends from 1996, the year of Boyer’s proposal to make engaged scholarship a core university mission, to January 2016, inclusively. We searched various databases: Academic Search Complete, Education Source, ERIC, FRANCIS, MEDLINE, PsycARTICLES, PsycCRITIQUES, PsycEXTRA, Psychology and Behavioral Sciences Collection, PsycINFO, Social Work Abstracts, SocINDEX via CINAHL Plus database, and Google Scholar.

Search terms were chosen to reflect the core concept. The search was conducted in January 2016 using a key-word combination: [public scholarship OR scholarship of engagement OR engaged scholarship]. Duplicate references were filtered out, and only English language articles were retained.

### Selecting studies

To be considered, studies had to have engaged scholarship as their main focus. We retained only primary studies that defined the concept theoretically, whether at the individual or institutional level, rather than empirical studies. The papers also had to be published in peer-reviewed journals.

**Fig 1:**
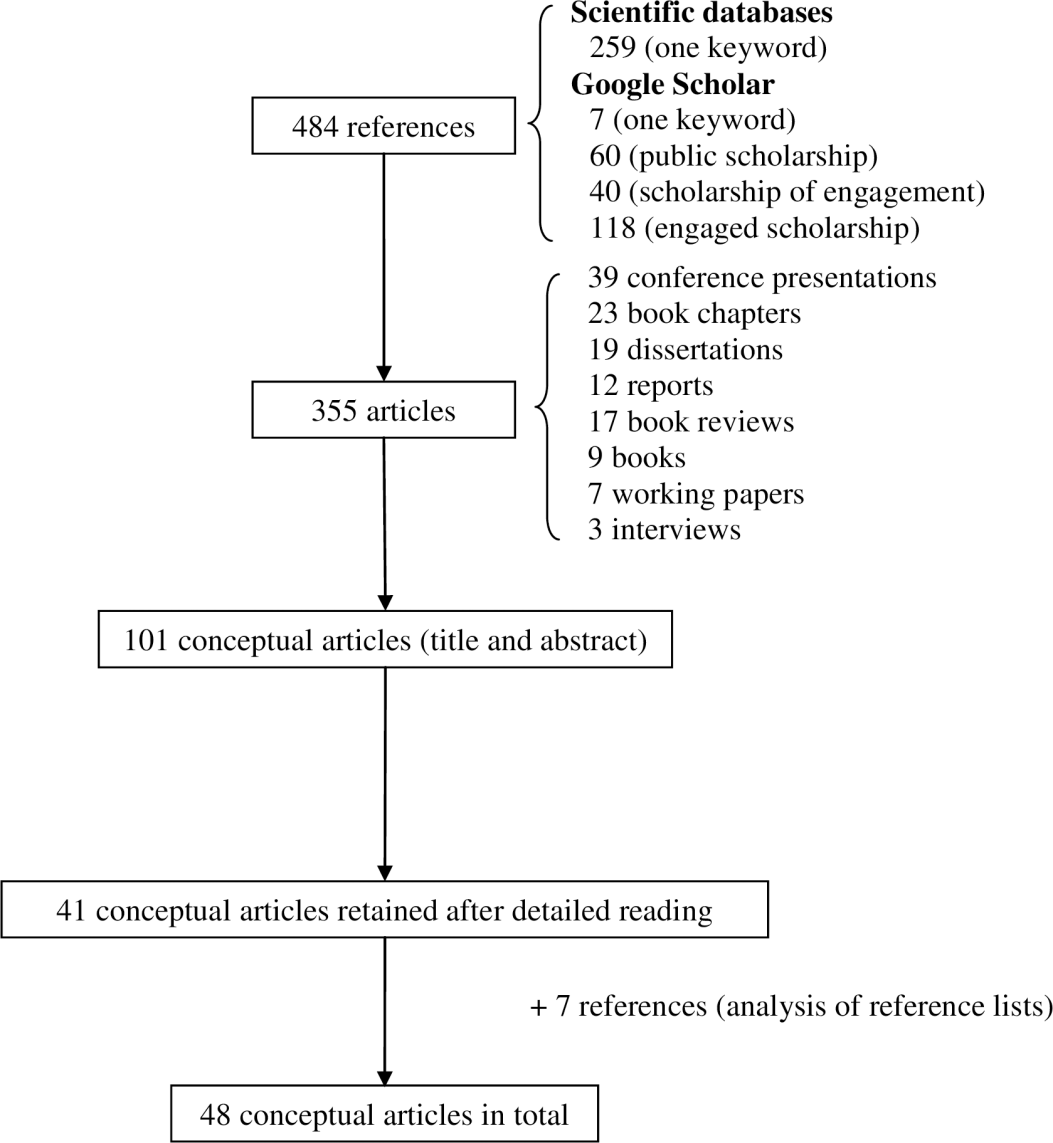
Article selection process.

The initial screening identified 484 references. News items, letters, editorials, book reviews, and articles appearing in newsletters or magazines rather than in peer-reviewed publications were then excluded. Articles with obviously irrelevant titles and abstracts were also excluded. We screened the full text of remaining articles to ensure they conceptually described engaged scholarship (Fig 1). References from relevant articles were then scanned to identify other papers not captured in the first screening.

### Charting the data

As shown in Fig 1, 48 papers were retained for analysis. Content was extracted and synthesized using a “thematic construction in order to present a narrative account of the existing literature” [11, p.27]. The articles retained were analyzed in three phases. First we performed an exploratory reading of each one. Then they were scanned for definitions of engaged scholarship. After this step, the three authors discussed the definitional elements found in the articles and together established the analysis criteria. Each paper retained was then read again and assessed on three criteria: values, principles, and process (individual, institutional).

Table 1 presents the distribution of the references by discipline, country, and year of publication. The increase in the annual number of publications since 1996 demonstrates the growing interest in this movement, particularly over the past five years. While the majority of publications are from the United States, other countries have become interested recently. Even though authors in many fields have written on the subject, the education sector is clearly dominant.

**Table 1:** Description of the references (n = 48).

	n (%)
**Year of publication**	
1996–1999	1 (2)
2000–2004	6 (13)
2005–2009	15 (31)
2010–2015^a^	26 (54)
**Country of publication**^**b**^	
USA	37 (77)
Australia	5 (10)
UK	3 (6)
Canada	2 (4)
Ireland	1 (2)
**Discipline**	
Education (or higher education)	26 (54)
Health sciences, public health and nursing	5 (10)
Business/management	2 (4)
Communication	2 (4)
Political sciences and public policy	2 (4)
Sociology	2 (4)
Community research	1 (2)
Ecology	1 (2)
Engineering	1 (2)
Human resources development	1 (2)
Leisure	1 (2)
Museology	1 (2)
Pharmacy	1 (2)
Psychology	1 (2)
Urban studies and planning	1 (2)

^a^ 2 retained references were published in 2015.

^b^ Before 2010, all references were from the USA.

To structure the positions and reflections emerging from the literature, to go beyond using engaged scholarship as an “umbrella term” [12], and to give meaning to what Sandmann [13] calls a “definitional anarchy”, we structured the analysis around the identification of values, principles, and processes. As shown in Table 2, we found two central values: social justice and citizenship. We also identified five core principles: high quality scholarship, reciprocity, identified community needs, democratization of knowledge, and boundary-crossing. Finally, we established two levels of engagement processes: individual (teaching, research, service) and institutional (mission, reward structure, logistical support, students). The results present in detail how these foundational elements are positioned in engaged scholarship.

**Table 2:** Overview of concepts found in the literature (n = 48).

	VALUES		PRINCIPLES							PROCESS				
								INDIVIDUAL			INSTITUTIONAL			
	Social justice	Citizenship	High quality scholarship	Reciprocity	Identified community needs	Democratization of knowledge	Boundary-crossing	Teaching	Research	Service	Mission	Reward structure	Logistical support	Students
Adshead, 2015								▪			▪			▪
Barge & Shockley-Zalabak, 2008							▪	▪				▪		
Barker, 2004	▪		▪	▪			▪		▪					
Bhattacharyya & Murji, 2013		▪				▪								
Bowen & Graham, 2013				▪										
Boyer, 1996								▪		▪				
Brazeau & Nemire, 2009								▪	▪	▪				
Bridger & Alter, 2006	▪	▪	▪	▪	▪									
Brown et al., 2003				▪		▪								
Burrage et al., 2005				▪				▪	▪			▪		
Calleson et al., 2005								▪	▪	▪	▪	▪		
Campbell, 2012		▪												
Checkoway, 2013		▪						▪	▪	▪	▪	▪	▪	
Colbeck & Wharton-Michael, 2006a							▪							
Colbeck & Wharton Michael, 2006b								▪	▪					
Colledge, 2014								▪				▪		▪
Cooper & Skipton, 2013							▪				▪	▪		
Cuthill & Brown, 2010		▪	▪	▪			▪				▪			
Cuthill et al., 2014	▪	▪	▪	▪	▪						▪			
Cuthill, 2010									▪					
DeLugan et al., 2014				▪					▪			▪		
Doberneck et al., 2010			▪						▪	▪				
Douglas, 2012											▪			
Dwight, 2008				▪										
Finkelstein, 2001				▪	▪		▪						▪	
Fitzgerald et al., 2012	▪		▪	▪			▪				▪		▪	
Franz, 2009				▪				▪	▪					
Furco, 2010			▪	▪			▪	▪	▪	▪	▪			
Gelmon et al., 2013				▪				▪	▪			▪		
Gibson, 2012											▪	▪		▪
Hartelius & Cherwitz, 2010		▪	▪											
Karp, 2012					▪									
McCormack, 2011									▪					
Mitchell & McDonald, 2012	▪	▪						▪						
Morton, 2013	▪	▪		▪	▪			▪			▪			
Paynter, 2014		▪	▪	▪										
Potter et al., 2009										▪		▪		
Ramaley, 2009		▪						▪	▪					
Sandmann, 2006							▪							
Sandmann, 2008				▪			▪	▪	▪					▪
Schneider, 2001								▪						
Schwab et al., 2014										▪				
Shackford-Bradley, 2013	▪	▪						▪						
Silka et al., 2013							▪							
Tsui, 2013									▪					
Van de Ven & Johnson, 2006			▪	▪	▪		▪		▪					
Vogelgesang et al., 2010														
Wood, 2003								▪						

## Results

In this section, we present the values and principles underlying engaged scholarship, as well as the processes put forward in the literature in which it is conceptualized.

### Values

Engaged scholarship is characterized by two core values: social justice and citizenship. These values are so important that Douglas [14] suggests the focus should not be on *how* faculty engage (activity), but rather on *why* they engage (value).

#### Social justice

While some authors position engaged scholarship squarely in the social justice arena [15, 16], many refer to social justice principles without being explicit. Some speak of the importance of fighting for equity and civil democracy by integrating vulnerable and marginalized populations into research and action [12, 17]. Ultimately, engaged scholarship should focus on individual and social well-being [18]. For several authors, faculty have a moral obligation to develop complementary relationships between scholarly achievement and the public good and to study public issues [12, 18–20], given that, especially in Canada, most research activities and universities are publicly funded.

#### Citizenship

Bridger and Alter [18] assert that faculty need to integrate their role as expert with their role as citizen. To maximize the impact of their work, these two roles must become inseparable—for example, in choosing to act as consultants in activities or projects that are outside the university but that can benefit from their expertise [18]. This requires “engaged faculty” to think and act as members of society [16, 21] in order to change the world for the better [19, 20, 22]. This broadening of faculty’s civic roles has led some to become “citizen-scholars” [23], while others have gone so far as to call this posture “activist scholarship” [24]. These activists see a need to reconsider how university work is positioned in order to unite the two worlds (the academy and the public sphere), going even so far as to propose that intellectual productions and political involvement should be joint efforts, so that they are mutually enriching [24]. In this respect, the engaged researcher acknowledges having a social accountability and civic responsibility to engage with the wider society—at local, national and international levels—on issues of public relevance [3, 15, 19, 20, 25, 26].

### Principles

Engaged scholarship also respects several principles. In our scoping review, five such principles emerged: 1) high-quality scholarship; 2) reciprocity; 3) identified community needs; 4) boundary-crossing; and 5) democratization of knowledge.

#### High-quality scholarship

The first principle is that of meeting the highest academic standards through high-quality scholarship [3, 18, 19]. Cuthill & Brown [3, p.130] assert that “quality within engaged scholarship is both academically defined and socially accountable”. Thus, while engagement requires a great sense of rigour [17, 25], it also calls for reconsidering the ways in which researchers conduct themselves, to ensure the value and relevance of research on both the social and academic levels [27]. For intellectual activities to be significant, it is important that the partners show flexibility and creativity [23, 28], in finding ways not only to produce knowledge in collaboration with actors from civil society, but also to communicate this knowledge to the public [17].

#### Reciprocity

Engaged faculty need to adopt reflective and iterative methods [3], in order to maximize their impact rather than their products [12]. To produce this impact, and especially to broaden and deepen the connections between the university and civil society [17], partnerships and collaboration are at the heart of engaged scholarship [19, 20, 29]. More particularly, partnerships should also include non-academic or practitioner partners, who often have a fuller understanding of complex societal issues [27, 30, 31]. Moreover, academic engagement considers creating such partnerships to be an integral part of academic functions [17]. Public involvement, the integration of different types of knowledge in knowledge production [3], and the creation of meaningful interactions are all recognized as critical factors in predicting research use [32]. This suggests the importance of reciprocity between the academy and civil society during not only the production of knowledge, but also its dissemination [12, 13, 17, 18, 33, 34]. It involves sharing knowledge and resources to produce sustainable and mutually beneficial outcomes for both communities and universities [13, 19, 25, 27, 35–37].

#### Identified community needs

In their work, engaged faculty address important civic issues or real societal problems [18]. For this, they need to adopt a perspective that is problem- rather than theory-driven [31]. They are called to be socially responsive [20] and to organize intellectual activities that are deeply rooted in practice [19].

#### Boundary-crossing

In contrast to their colleagues who follow more traditional career paths, engaged faculty do not conceive of their research programs in accordance with the “intellectual agenda” of a particular discipline [38]. Engaged scholarship fundamentally involves a multi-inter-transdisciplinary approach [3, 31, 39]. It assumes an interaction across disciplines and relevant sectors. Moreover, engaged scholarship must overcome disciplinary boundaries [3, 17]. In this sense, it is a boundary-crossing scholarship [40, 41], because:

“…*putting theory and practice in relationship with each other is not an intellectual cognitive activity that can be constructed in one’s head; rather*, *it is an embodied relational activity that necessitates bringing members of scholarly and practitioner communities into conversation with one another*. *Engaged scholarship privileges the diversity of perspectives that theorists and practitioners bring to making sense of a problem and honors their unique knowledge and expertise as valid*.*”* [42, p.252–253].

Faculty work has long been departmentalized by discipline, and it is becoming increasingly subdivided into distinct and separate teaching, research, and service tasks [43]. Far from being mutually exclusive and segmented, engaged scholarship is focused on integrating teaching, research, and service [12, 13, 27, 31, 40]. In this view, engagement requires putting teaching and service on a par with research [12]. Moreover, it requires integrating their academic activities into a coherent whole [43, 44]. This means that teaching and service activities should be based on advancing one’s research, and that teaching should be seen as a form of service to the community [44]. Far from taking on extra workload, by planning integrated activities that combine different academic functions and address several objectives simultaneously, engaged faculty optimize the impact of their work [44].

#### Democratization of knowledge

Engaged scholarship argues for the democratization of scientific knowledge and its accessibility for all [33]. It also questions the assumption that the academy holds a monopoly over knowledge production, and instead sees the academy as an important locus for debate around decentralizing and rethinking knowledge production [24].

### Engagement process

The values and principles presented above are embodied at two levels of academic life: at the individual level in various activities conducted by the engaged faculty, and at the institutional level in university or research centre programming.

#### Individual level

The role of faculty consists in three interrelated areas of activity: teaching, research, and service. Engagement finds expression not only in each of these separate academic functions, but also in their integration.

*Engaged teaching* is the most developed of the three academic functions in the literature, especially in the United States. It equates to transmitting, transforming, and extending knowledge and bringing about change in learning with various audiences through either formal or informal arrangements [21, 34]. Despite this, the literature concentrates primarily on teaching university students. In this context, engaged teaching views the community as a landscape for strengthening students’ discovery and learning, in which they think and act on local as well as global issues of real importance [9, 27, 44]. It is primarily intended to facilitate students’ ability to integrate theory and practice and engage in praxis [42, 45]. This implies that students should be broadly prepared with deep knowledge and the professional skills required to be successful not only in the workplace [46], but also within the existing social, political, and economic institutions and relations of today and tomorrow [47]. In other words, engaged teaching educates students to live as responsible citizens, mobilize multiple forms of knowledge to make good decisions, and use their capacities to contribute to public good [21, 26, 37, 47]. To achieve this, university curricula combine the acquisition of traditional knowledge with concrete actions, so that the teaching model is anchored in reality and more active (such as hands-on learning) [35, 45, 48]. Engaged faculty pay special attention to how they teach, and the teaching approaches most often cited are service-learning [13, 15, 16, 20, 27, 35, 49, 50] and experiential learning [16, 20]. Schneider [48] defines service-learning (also called community-based learning) as students’ direct involvement:

*“…with societal issues and with groups seeking to solve problems and improve the quality of life for themselves and others*. *Again*, *the instructor's role is to provide social*, *moral*, *and technical context to help students generalize from the particular*, *connect scholarship with practice*, *and articulate grounds for commitment and action*. *Students establish* ***new*** *and reciprocal relationships with community leaders*, *and they come to recognize the legitimacy of experiences and perspectives very different from their own*.*”* [48, Hands-on pedagogies: para.3]

Schneider [48] also defines experiential learning as:

*“…direct experience in field settings*, *with open-ended problems*, *projects*, *and challenges*. *The instructor helps the students*, *either individually or as a group*, *learn to process their experience*, *put it in a context of general principle—practical*, *intellectual*, *and ethical—and rethink their content learning in light of the field experience*. *The boundaries between theory and practice are blurred*, *with practice accepted as a legitimate source both of knowledge and challenge to reigning theories*.” [48, Hands-on pedagogies: para.2].

Other engaged teaching approaches were also identified: project-based learning [48], integrative learning [48], relational learning [48], and internships [27]. Those activities can take place in a campus setting or off campus where learning has meaningful consequences that can influence the thinking and the lives of others [26]. Whatever the teaching approach, creating reflective spaces is highly recommended to maximize learning from these experiences [42].

*Engaged research* incorporates reciprocal civic engagement practices into the discovery, development, and mobilization of knowledge to the mutual benefit of community and academic interests [17, 34, 36, 38]. Doberneck and colleagues [28, p.20] go further, asserting that it also includes creative activities, defined as “original creations of artistic, literary, fine, performing, or applied arts and other expressions or activities of creative disciplines or fields that are made available to or generated in collaboration with a public (nonuniversity) audience.” However, engaged research must be systematic and rigorous [51] and, as such, must be based on the current state of knowledge, and results need to be disseminated in publications so they can be critically reviewed and debated among peers [37]. However, the relevance of engaged research is not limited to developing science based on the latest theories and methods [52]. More essential is the integration of theory and practice, as well as the inclusion of community partners as active contributors to identify clear goals and research questions that address a real-world issue [26, 27, 44, 52]. Thus, besides designing and conducting the best research, in scientific terms, researchers have to work on issues that are useful and meaningful to the community, so that the results ultimately benefit that community [26, 27, 52]. To do so, they use various research approaches: community-based participatory research or community-based research [13, 27, 35, 49, 50], participatory research or participatory action research [13, 27, 51, 53], and applied research [13].

*Engaged service* is defined as the application of a professor’s expertise and scientific or professional knowledge to address specific issues for the benefit of policy makers, public officials, agencies, organizations, professionals, and civil society [21, 54, 55]. As such, engaged service takes the form of a variety of activities, such as advocacy [50], outreach [50], technical assistance [28], and expert testimony or legal advice [28]. For Schwab and colleagues [55], service should be at the heart of an academic career. They suggest that “rather than considering service to be a poor stepchild to research and teaching [activities], service should be (…) the driving force behind good teaching, student learning, and scholarship” [55, p.26].

#### Institutional level

Brown and colleagues [33] explain that faculty and scholar engagement depends on context, which they describe in terms of three levels: organizational, institutional, and external (political, economic, social, cultural and technological context). The traditions and values associated with these different levels of influence play a determining role in impeding or supporting activities of engagement among faculty and students [42]. “This is not to say that organizations drive individual behavior, but rather that faculty members’ personal values play out differently depending to some extent on the organizational environment” [56, p.441]. Thus, institutionalization of engaged scholarship is key to its success [57]. The institutional-level conceptualization of the engagement process encompasses four dimensions: mission, reward structure, logistical support, and students.

*Mission*. For an engaged university, it is essential not only to support research that responds to the needs of local communities and helps those communities, but also to form generations of students who are prepared to contribute positively to the world around them [12, p.15]. Yet, “over time (…), institutions have developed multiple purposes and, in so doing, de-emphasized their civic mission” [21, p.8]. The university culture has thus, over time, generally discouraged academic engagement. Faculty “are led to believe that engaged scholarship is not central to their roles, that there are few rewards for this work, and that it might even jeopardize their careers in the university” [21, p.13]. This is extremely problematic, given that many universities and research projects are publicly funded and, as such, have a civic duty to engage with the wider society—at local, national, and international levels—on issues of public relevance [19]. The “civic orientation” must be exemplified by the institution’s research, teaching, and service mission, and community engagement must be at the heart of this mission [14, 20, 21, 27, 40, 45, 58]. It needs to be seen as a critical tool for achieving the university’s higher purpose [14]. In fact, rather than being limited to the service sphere, community engagement should be seen as an essential component of high-level teaching and research [27]. To achieve this will require a profound transformation of university culture [14, 50]. Such a transformation will require not only that every faculty member embodies engagement [14], but also that institutional leaders support the implementation of engagement-oriented missions [3]. This way of thinking will inevitably lead to a new scholarship [14].

*Reward structure*. Faculty’s work has the greatest value when aligned with the institution’s missions and strategic priorities [50, 53]. An institution’s promotion or tenure guidelines are one of the strongest expressions of its priorities and values [37]. As such, the current definition of academic prestige and the publish-or-perish culture are the greatest impediments to engagement [42]. They lead “faculty members to view as time-wasters their important responsibilities of teaching and student learning, and service in the school and towards community stakeholders” [40, p.63]. University leaders thus have to modify these norms to shape a new culture that will make the university a place that supports engagement and values connections with the community [35]. Institutions committed to engaged scholarship must establish mechanisms that recognize and reward engaged scholarship practices [36]. This can be achieved through recruitment processes and promotion and tenure criteria that take into account the multidisciplinary and engaged dimensions of faculty’s work [37, 42, 46, 50, 53]. If these aspects are not recognized through the review, promotion, and tenure process, faculty could be reluctant to engage [36]. It is important that those engaging in teaching, research, and service associated with engaged scholarship feel that their contributions are valued by the university, the college, and the department [46]. To feel so, faculty should be rewarded for their work, including drawing upon their expertise (professional service, public work, and/or community-based action research or public scholarship) for the benefit of society as an integral part of their role [21, 57].

*Logistical support*. The role of institutional support is not negligible; it is decisive in helping faculty adequately respond to growing demands that they work actively for the public good [55]. To this end, universities need to introduce processes that can facilitate relationships with a variety of partners, whether within the university (between disciplines and departments) or outside it (with other universities and non-university partners) [21]. To help those collaborations, institutions may fund and create an administrative team to support engagement activities [12, 27].

*Support to students*. Some universities offer integrated programs or organize specific projects geared toward developing engagement-oriented skills and interests in organizations outside the university [45]. Students need support for engagement and involvement in the community; this includes engagement opportunities available to students as well as incentives and rewards for those engaged [57]. Moreover, integrating engaged scholarship into graduate education may encourage students to become, themselves, future engaged professionals [13].

### Engaged scholarship schema

This scoping review leads us to define engaged scholarship as a true academic posture, rooted in values of social justice and citizenship, that prompts academics and universities, in their roles of teaching, research, and service to society, to work in ways that will build mutually beneficial and reciprocal bridges between university activity and civil society.

To accomplish this, faculty are uniquely positioned at the heart of the engagement process, at the interface between both worlds, with three main functions: teaching, research, and service. To foster connections with society, faculty must adhere to five principles: high-quality scholarship, reciprocity, identified community needs, democratization of knowledge, and boundary-crossing. These principles will be applied in faculty’s ways of doing research, investing in community service, and teaching. As teachers, faculty are expected to play a key role in raising students’ awareness and encouraging them in building bridges between the university and society, science and practice, and research and action. While students are important actors, the university as an institution plays a major role in implementing engaged scholarship. In fact, it can greatly facilitate it by providing a clear mission, a structure that recognizes and rewards engagement activities, logistical support for engagement projects, and support for students who wish to become engaged. These core elements in the conceptualization of engaged scholarship make it an academic posture. Indeed, it is not a strategy to be adopted and applied in particular circumstances, as would be knowledge transfer activities, for example. Rather, it is their way of being and of practising their vocation—that is, how they view the purposes of their work, the methods and frameworks used, as well as the values they hold [59]—that makes professors into engaged faculty.

**Fig 2:**
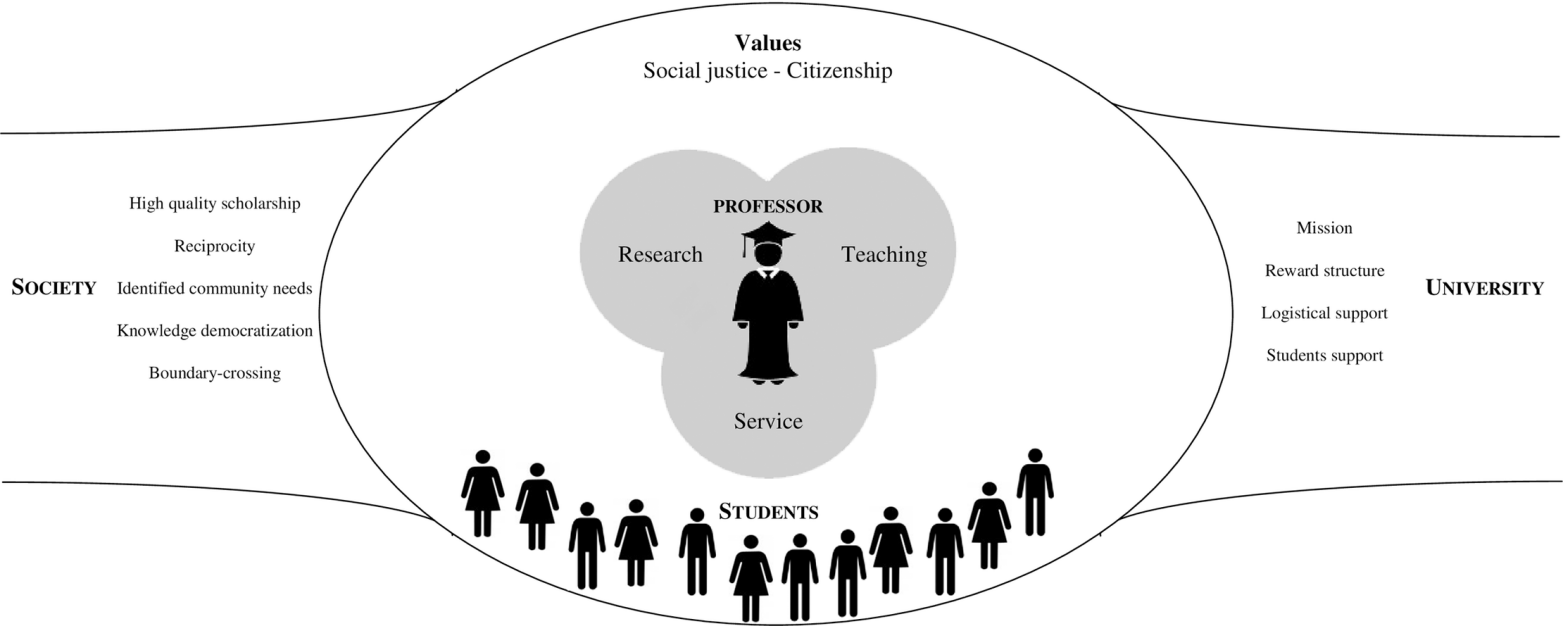
Engaged scholarship schema.

Fig 2 summarizes and illustrates the links between the key elements that define engaged scholarship.

## Discussion

Our analysis of the conceptual literature on engaged scholarship, and more specifically, on its underlying values and principles, and on its implications at the individual and institutional levels, sheds new light on this movement. Engaged scholarship is a new academic paradigm that affects not only the researcher’s role, but also those of the student and the university in society. In fact, recent years have seen significant changes in the academic environment. It is now widely recognized that, for faculty, working in isolation has become outdated and collaborating with other settings has become essential. As such, new streams are emerging that offer alternatives to traditional scientific approaches. Joining the engaged scholarship movement means voluntarily abandoning a paradigm of objective, neutral, and apolitical science for an engaged paradigm, in which problems under study are presented in new ways and innovative solutions are legitimized. Calling into question faculty’s neutrality and objectivity [17] is, in fact, a reaction to the more traditional post-positivist perspective. Therefore, “[community-engaged scholarship] reflects a differing epistemological basis and a wider set of values, goals, skills, and results” [40, p.65]. It suggests that the faculty role is not limited to knowledge production but expands to becoming “actors” of change who participate actively in creative intellectual activities with various stakeholders [21]. This unusual position requires engaging in knowledge production and building partnerships that foster knowledge and resource exchanges with civil society with a view to democratizing knowledge [24, 42]. It thus involves broadening academic functions [60] and supposes a decentralized expertise outside universities to formulate solutions for reducing the gap between scientific knowledge (university) and practice (civil society). To do so, “faculty members can create knowledge that contributes to civic development; teach and train people in areas of civic expertise; aggregate knowledge to make it more useful to civic agencies; disseminate knowledge to broad public and professional audiences; [and] advocate on issues” [21, p.12]. Engaged faculty seek actively to contribute to the common good through a variety of means that fall within their researcher and professor roles, but also their role of citizen, which they consider intrinsic to the others.

While useful for conceptualizing academic engagement, this scoping review nevertheless has certain limitations. As the analysis was focused on published articles in scientific journals, reports, books and monographs were excluded. Such documentation could provide complementary information and would warrant further analysis in an exhaustive literature review.

## Conclusion

Engaged scholarship appears to represent a turning point in what it means to be an academic today. This scoping review of the literature on engaged scholarship over the past two decades shows that the movement is not anecdotal, and that it is based on solid, coherent, and complementary foundations. Our analysis clarified the roles of faculty, students, and the university in society, the values underlying them, and the principles that support not only teaching and research, but also service activities. Our analysis indicates that this posture relies on institutional support in various forms (mission, reward structure, logistical support, support to students) to facilitate this role, to give greater exposure to engagement activities, and especially to ensure that faculty who adopt this posture are not penalized. This review has raised other questions that merit closer examination. Is it possible for faculty to adopt an engaged posture in a non-supportive environment? Can one become an engaged researcher without institutional support, and if so, at what cost? Should engaged scholarship be supported by a community? If so, which one—that of the society partners, or a community of faculty? Our analysis provides a clearer picture of the actors’ roles and responsibilities. We see that engaged faculty and students are the essential link between an institution—the university—and civil society. The interfaces between these two poles call for further exploration. Beyond discourse or theoretical principles, it would be important to identify the most promising practices, at both the institutional and individual levels, to actually achieve the ultimate objective, which is to influence and contribute to the well-being of our societies. Our contribution here has been to offer a conceptualization of this growing movement of engaged scholarship as a foundation for exploring its meaning in greater depth, analyzing its implementation in academic contexts, and further examining its specific features in comparison with other emerging movements aimed at giving scientific knowledge a greater role in building our society.
